# Pancreatic cancer cell–derived exosomal microRNA‐27a promotes angiogenesis of human microvascular endothelial cells in pancreatic cancer via BTG2

**DOI:** 10.1111/jcmm.14766

**Published:** 2019-11-13

**Authors:** Dan Shang, Chao Xie, Jin Hu, Jinru Tan, Yufeng Yuan, Zhisu Liu, Zhiyong Yang

**Affiliations:** ^1^ Department of Vascular Surgery Union Hospital Tongji Medical College Huazhong University of Science and Technology Wuhan China; ^2^ Department of Hepatobiliary and Pancreatic Surgery Zhongnan Hospital of Wuhan University Wuhan China; ^3^ Pancreatic Surgery Center Zhongnan Hospital of Wuhan University Wuhan China; ^4^ Department of Pancreatic Surgery Union Hospital Tongji Medical College Huazhong University of Science and Technology Wuhan China

**Keywords:** angiogenesis, human microvascular endothelial cell, microRNA‐27a, pancreatic cancer

## Abstract

Pancreatic cancer (PC) remains a primary cause of cancer‐related deaths worldwide. Existing literature has highlighted the oncogenic role of microRNA‐27a (miR‐27a) in multiple cancers. Hence, the current study aimed to clarify the potential therapeutic role of PC cell–derived exosomal miR‐27a in human microvascular endothelial cell (HMVEC) angiogenesis in PC. Initially, differentially expressed genes (DEGs) and miRs related to PC were identified by microarray analysis. Microarray analysis provided data predicting the interaction between miR‐27a and BTG2 in PC, which was further verified by the elevation or depletion of miR‐27a. Next, the expression of miR‐27a and BTG2 in the PC tissues was quantified. HMVECs were exposed to exosomes derived from PC cell line PANC‐1 to investigate the effects associated with PC cell–derived exosomes carrying miR‐27a on HMVEC proliferation, invasion and angiogenesis. Finally, the effect of miR‐27a on tumorigenesis and microvessel density (MVD) was analysed after xenograft tumour inoculation in nude mice. Our results revealed that miR‐27a was highly expressed, while BTG2 was poorly expressed in both PC tissues and cell lines. miR‐27a targeted BTG2. Moreover, miR‐27a silencing inhibited PC cell proliferation and invasion, and promoted apoptosis through the elevation of BTG2. The in vitro assays revealed that PC cell–derived exosomes carrying miR‐27a stimulated HMVEC proliferation, invasion and angiogenesis, while this effect was reversed in the HMVECs cultured with medium containing GW4869‐treated PANC‐1 cells. Furthermore, *i*n *vivo* experiment revealed that miR‐27a knockdown suppressed tumorigenesis and MVD. Taken together, cell‐derived exosomes carrying miR‐27a promotes HMVEC angiogenesis via BTG2 in PC.

## 1 INTRODUCTION

Pancreatic cancer (PC) ranks as the seventh leading cause of cancer‐related death worldwide, accounting for approximately 4% of all cancer cases with about 330,000 deaths per year.^1^ PC has also been highlighted as one of the most aggressive malignancies in digestive system, due largely to late diagnoses, high rate of mortality, as well as an increased potential of metastasis and malignancy.^2^ Endothelial cells are a type of heterogeneous cell cluster located in the microvascular capillary beds of various organs, among which human microvascular endothelial cells (HMVECs) are unique yet unrecognizable in phenotype, function and structure.^3^ Angiogenesis has been widely implicated in the process of tumour development.^4^ Moreover, exosomes derived from PC cells trigger the acceleration of angiogenesis in HMVECs via a dynamin‐dependent endocytosis process.^5^

Exosomes are extracellular vesicles with a size of 40‐150 nm play a functional role in PC therapy.^6^ Exosomes secreted by tumours can assume the role of carriers to deliver microRNAs (miRs) into recipient cells in the cancer microenvironment, which has been reported to enhance tumour metastasis and invasion.^7^ Both miRs and miR exosomes have been speculated to be promising PC therapeutic targets through the protein transfer‐induced target cell reprogramme and binding‐triggered signal transduction.^8^ miRs are a group of short non‐coding RNA molecules with the length of 18‐22 nucleotides, which regulate genes associated with cell apoptosis, differentiation and neoplastic transformation.^9^ The abnormal expression of miRs has been implicated in the development and progression of a large array of cancers, including that of PC.^10^ Additionally, the targeting of miR‐27a by the anticancer agent 2‐cyano‐3,12‐dioxooleana‐1,9‐dien‐28‐oic acid (CDDO) has been reported to diminish its expression leading to an inhibition of tumour cell growth through anti‐angiogenic responses.^11^ Existing literature has indicated that miR‐27a directly targets B‐cell translocation gene 2 (BTG2) in gastric cancer cells.^12^ BTG2 is an immediate early response protein that has been shown to play a role in DNA damage repair, antiproliferation, cell apoptosis and differentiation, whose elevated level has been identified in the majority of normal tissues, including the pancreas.^13^ BTG2 is regarded as a tumour inhibitor in pancreatic ductal adenocarcinoma and has been implicated in the progression and growth of PC under the control of miR‐21, miR‐23a and miR‐27a.^14^ Based on the aforementioned data, we examined the hypothesis that PC cell–derived exosomes carrying miR‐27a influence the angiogenesis of HMVEC through BTG2 in PC. Hence, this study indented to investigate the underlying molecular mechanisms associated with the progression of PC to elucidate more effective therapeutic strategies for PC patients through the use of PC cell–derived exosomes co‐cultured with HMVECs.

## 2 MATERIALS AND METHODS

### 2.1 Ethics statement

The study was performed in strict accordance with the recommendations of the Guide for the Care and Use of Laboratory Animals of the National Institutes of Health. The protocols were approved by the Institutional Animal Care and Use Committee of Union Hospital, Tongji Medical College, Huazhong University of Science and Technology. The experiment was approved by the Ethics Committee of Union Hospital, Tongji Medical College, Huazhong University of Science and Technology. Written informed consent was obtained from all participating patients in the study prior to enrolment.

### 2.2 Microarray‐based gene expression profiling

Differentially expressed genes (DEGs) were screened by downloading PC‐related microarray data (http://www.ncbi.nlm.nih.gov/geo/query/acc.cgi?acc=GSE22780, http://www.ncbi.nlm.nih.gov/geo/query/acc.cgi?acc=GSE16515, http://www.ncbi.nlm.nih.gov/geo/query/acc.cgi?acc=GSE32676 and http://www.ncbi.nlm.nih.gov/geo/query/acc.cgi?acc=GSE91035) from the Gene Expression Omnibus (GEO) database (https://www.ncbi.nlm.nih.gov/geo/) (Table 1). The pre‐treatment standardization on gene expression data was performed with the Affy package of R language,^15^ and the limma package was applied to screen the DEGs with a heat map subsequently plotted.^16^ Gene Expression Profiling Interactive Analysis (GEPIA; http://gepia.cancer-pku.cn/) was utilized for the verification of DEG expression and the analysis of the correlation between gene expression and survival of patients. jvenn (http://jvenn.toulouse.inra.fr/app/example.html) tool was employed for comparison of DEGs from four microarray data and to plot Venn map. Five miR‐mRNA interaction prediction databases, miRWalk (http://mirwalk.umm.uni-heidelberg.de/), TargetScan (http://www.targetscan.org/vert_71/), mirDIP (http://ophid.utoronto.ca/mirDIP/), DIANA (http://diana.imis.athena-innovation.gr/DianaTools/index.php?r=microT_CDS/index) and starBase (http://starbase.sysu.edu.cn/mirMrna.php), were employed to predict the miRs that regulate the DEGs, the results of which were compared using jvenn. In addition, two additional PC‐related miR data sets (http://www.ncbi.nlm.nih.gov/geo/query/acc.cgi?acc=GSE41369 and http://www.ncbi.nlm.nih.gov/geo/query/acc.cgi?acc=GSE28955) were analysed to screen differentially expressed miRs.

**Table 1:** PC‐related gene and miRNA expression chips

Accession	Platform	Organism	Gene/miRNA	Sample
http://www.ncbi.nlm.nih.gov/geo/query/acc.cgi?acc=GSE22780	GPL570	Homo sapiens	Gene	8 pancreatic tumour tissues and 8 adjacent normal tissues
http://www.ncbi.nlm.nih.gov/geo/query/acc.cgi?acc=GSE16515	GPL570	Homo sapiens	Gene	36 pancreatic cancer tumour samples and 16 normal samples
http://www.ncbi.nlm.nih.gov/geo/query/acc.cgi?acc=GSE32676	GPL570	Homo sapiens	Gene	25 pancreatic cancer tumours and 7 non‐malignant pancreas samples
http://www.ncbi.nlm.nih.gov/geo/query/acc.cgi?acc=GSE91035	GPL22763	Homo sapiens	Gene	8 normal pancreatic tissue and 27 pancreatic cancer tissues
http://www.ncbi.nlm.nih.gov/geo/query/acc.cgi?acc=GSE41369	GPL16142	Homo sapiens	miRNA	9 normal pancreas and 9 pancreatic ductal adenocarcinoma
http://www.ncbi.nlm.nih.gov/geo/query/acc.cgi?acc=GSE28955	GPL6955	Homo sapiens	miRNA	16 pancreatic cancer cell lines and 7 normal pancreatic samples

Abbreviation: PC, pancreatic cancer.

### 2.3 Study volunteer

Surgical specimens were collected from 60 PC patients who had been admitted to Department of Pancreatic Surgery, Union Hospital, Tongji Medical College, Huazhong University of Science and Technology, between June 2015 and June 2017. The control specimens included pancreatic tissues collected from 23 patients diagnosed with acute and chronic pancreatitis (15 cases of acute pancreatitis and 8 cases of chronic pancreatitis). Some of the specimens post‐operation were promptly frozen in liquid nitrogen at −80°C, and the remaining ones were embedded in paraffin, fixed and sectioned after being soaked in 4% formalin. All clinical data are shown in Table S1.

### 2.4 Immunohistochemistry

The paraffin‐embedded sections of tissue specimens were dehydrated using gradient ethanol and treated with water‐bath repair in antigen retrieval buffer. The sections were then incubated with normal goat serum confining liquid (C‐0005, Shanghai Haoran Biological Technology Co Ltd) at room temperature for 20 minutes. The sections were subsequently probed with primary rabbit anti‐human antibody against BTG2 (ab85051, 1:300, Abcam Inc) at 4°C overnight and with secondary goat anti‐rabbit immunoglobulin G (IgG) antibody (ab6785, 1:1000, Abcam Inc) at 37°C for 20 minutes. The sections were then allowed to react with horseradish peroxidase (HRP)‐labelled streptavidin protein working solution (0343‐10000U, Imunbio) at 37°C for 20 minutes, developed with diaminobenzidine (DAB; ST033, Whiga Biosmart Co Ltd), counterstained with haematoxylin (PT001, Shanghai Bogoo Biological Technology Co Ltd) for 1 minute and treated with 1% ammonium hydroxide. Finally, the sections were dehydrated by gradient ethanol, cleared with xylene, sealed with neutral balsam, analysed and photographed under a microscope.^17^

### 2.5 Dual‐luciferase reporter gene assay

The binding site between BTG2 and miR‐27a was analysed using the biological prediction website (https://cm.jefferson.edu/rna22/Interactive/), which was then verified through the application of a dual‐luciferase reporter gene assay. A BTG2 dual‐luciferase reporter gene vector and mutant (MUT) on binding sites miR‐27a and BTG2 were constructed, namely PGLO‐BTG2 wild‐type (WT) and PGLO‐BTG2 MUT. The aforementioned two reporter plasmids were respectively cotransfected with miR‐27a mimic and negative control (NC) plasmids into human PC cells. Dual‐Luciferase^®^ Reporter Assay System (E1910, Promega Corporation) was applied for luciferase activity detection.

### 2.6 Cell treatment

The human pancreatic ductal cell line H6c7, PC cell lines (SW1990, Capan‐1, BxPc‐3 and PANC‐1) and microvascular endothelial cell line HMEC‐1 were all purchased from Shanghai Institute of Cellular Research, Chinese Academy of Sciences. The cells were cultured with Roswell Park Memorial Institute (RPMI) 1640 medium containing 10% foetal bovine serum (FBS), followed by the addition of penicillin‐streptomycin solution at ratio of 1:1. The cells were then seeded into a 6‐well plate at the density of 3 × 10^5^ cells/well. Once the cells reached 70%‐80% confluence, the cells at the logarithmic growth phase were collected for subsequent experimentation.

The PC cell lines at the logarithmic growth phase were seeded into a 6‐well plate at a density of 4 × 10^5^ cells/well. The cells were cultured for 48 hours, after which further culture was conducted in RPMI 1640 medium (Santa Cruz Biotechnology Inc) containing 10% FBS for 24‐48 hours. All transfection sequences and plasmids were purchased from Shanghai GenePharma Co Ltd following transfection with miR‐27a mimic, miR‐27a inhibitor and BTG2 plasmids in accordance with the instructions of the Lipofectamine 2000 (11668‐019, Invitrogen Inc).

### 2.7 5‐Ethynyl‐2’‐deoxyuridine (EdU)

Cells at the logarithmic growth phase were seeded into a 96‐well plate at the density of 2 × 10^3^‐4 × 10^4^ cells/well. Transfection was performed 24 hours after the cells had adhered to the wall, with 3 duplicate wells set for each treatment. After 48 hours of transfection, cells were treated with EdU labelling^18^ with antifluorescence quenching mounting medium added at a density of 100 μL per well. The images were photographed under a fluorescence microscope. Cells with red‐stained nucleus were regarded as the positively labelled cells. Finally, 3 power fields were randomly selected to count the number of positive and negative cells under the guidance of a microscope.

### 2.8 Flow cytometry

Annexin V‐fluorescein isothiocyanate/propidium iodide (FITC/PI) double staining kit (556547, Shanghai Shoujia Biotechnology Co Ltd) was employed to detect cell apoptosis following a 24‐hours period of transfection. The cells were centrifuged at 715 *g* for 5 minutes at room temperature, followed by re‐suspension with pre‐cooled 1× phosphate‐buffered saline (PBS). After further centrifugation was performed at 715 *g* for 5‐10 minutes, the cells were suspended with 300 µL of 1× binding buffer. The cells were incubated at room temperature under dark conditions for 15 minutes following a uniform mixture with 5 µL annexin V‐FITC. The cells were then added with 5 µL PI and ice‐bathed under conditions void of light for 5 minutes. Finally, FITC was subsequently detected at an excitation wavelength of 480 nm and 530 nm with PI detected at an excitation wavelength of more than 575 nm using a flow cytometer (Cube 6, Partec).

### 2.9 Transwell assay

The pre‐cooled Matrigel was diluted using serum‐free Dulbecco's modified Eagle's medium (DMEM; 40111ES08, Shanghai Yeasen Biological Technology Co Ltd) at a ratio of 1:2 and settled into the apical chamber of a Transwell chamber (3413, Beijing Unique Biotechnology Co Ltd), followed by incubation for 4‐5 hours for solidification. Next, the transfected cells were diluted with 100 μL serum‐free medium in order to prepare cell suspension at a concentration of 1 × 10^6^ cells/mL, which was then seeded into the apical chamber. Next, 500 μL of DMEM containing 20% FBS was added into the basolateral chamber, with 3 duplicate wells prepared for each treatment. After 24‐hours incubation, the Transwell chambers were fixed with 5% glutaraldehyde at 4℃, stained with 0.1% crystal violet for 5 minutes and observed under an inverted fluorescence microscope (TE2000, Nikon). Five fields were randomly selected to acquire images, with the mean value calculated as the number of cells crossing the chambers.

### 2.10 Exosome extraction and identification

The PANC‐1 cells were seeded into a 6‐well plate at the density of 1 × 10^5^ cells/well with the H6c7 cells employed as the control. After the cells had adhered to the wall overnight, the exosome serum was renewed for an additional 48‐hours culture. A total of 5 mL supernatant was collected from every 3 duplicate wells for exosome extraction in strict accordance with the instructions of the ExoQuick‐TC kit (ExoQuick‐TC, Shanghai Shanran Biotechnology Co Ltd). Afterwards, 30 μL exosomes were added on the copper wire mesh, allowed to stand for 1 minute, counterstained with 30 μL Salkowski's solution (pH = 6.8) at room temperature for 5 minutes and photographed under a transmission electron microscope (TEM).

The magnetic beads as well as the CD63 antibody were incubated with 50 μL PBS for 30 minutes at 37℃ (total volume of 400 μL) and vibrated on a shaking table for 24 hours. Sample blockade was subsequently conducted with FBS at 4℃ for 5 minutes. After 4 consecutive cycles of the aforementioned procedures, the exosomes extracted at 4℃ were incubated with magnetic beads for 24 hours. Later, each part was added with CD63‐polyethylene (PE) antibody and incubated at room temperature for 30 minutes as per the provided antibody instructions. The cells that were not added with an antibody were regarded as the blank control, and the PE‐labelled anti‐human IgG was regarded as the isotype control. The samples were then loaded followed by detection using a Guava easyCyte™ flow cytometry system. Reverse transcription‐quantitative polymerase chain reaction (RT‐qPCR) was performed in order to determine the expression of miR‐27a in the exosomes.

### 2.11 Co‐culture of exosomes and HMVECs

The exosomes dissolved with PBS were mixed with Exo‐Red (EXOR100A‐1, Chang Zhou Bei Yuan Xin Bio‐Technique Co Ltd) at a ratio of 10:1, followed by incubation for 10 minutes. After reaction termination with 100 μL stop buffer, exosomes were further incubated at 4℃ for 30 minutes and centrifuged at 35 068 *g* for 3 minutes. Fluorescence‐labelled exosomes were re‐suspended with 200 μL PBS and co‐cultured with green fluorescent protein (GFP)‐labelled HMEC‐1 cells which had been inoculated into a 24‐well plate. The HMEC‐1 cells were then either subjected to incubation with exosome‐free PBS (control), conditioned media collected from PANC‐1 cells treated with GW4869 (Exo‐depl), exosomes secreted by PANC‐1 cells (PANC‐1‐exo) and exosomes secreted by H6c7 cells (H6c7‐exo), or transfected with miR‐27a mimic. After incubation, the HMEC‐1 was washed 3 times with PBS and analysed under an inverted fluorescence microscope, after which the expression of miR‐27a was determined using RT‐qPCR.

### 2.12 Angiogenesis assay

Matrigel (356234, Shanghai Shanran Biotechnology Co Ltd) was dissolved into yellow colloidal liquid. Next, a pre‐cooled micropipette was used for a rapid transfer of 70 μL yellow colloidal liquid (0.5 mmol/L) into a pre‐cooled 96‐well plate. After 48 hours of transfection, the cells were starved without serum for 1 hours and re‐suspended in DMEM to make cell suspension. Next, the cell suspension (1 × 10^5^ cells/mL) was seeded into the culture wells settled with Matrigel. Each well was added with the corresponding cell culture medium, with 3 duplicate wells set for each treatment. The plate was subsequently incubated for 18 hours, after which photographs were obtained under the guidance of a Leica Inverted Phase Contrast Microscope. The number of complete capillary lumen surrounded by cells was calculated under a 100‐fold microscope using the Image‐Pro Plus (version 6.0), with at least 3 fields counted in each group.

### 2.13 RT‐qPCR

Total RNA was extracted from the tissues or cells in strict accordance with instructions on the TRIzol Kit (15596‐018, Beijing Solarbio Life Sciences Co Ltd). All primers (Table 2) were synthesized by Takara Biotechnology Co Ltd. Reverse transcription was performed based on the one‐step method in line with the instructions of the miRNA (D1801, HaiGene Co Ltd) and cDNA Reverse Transcription Kits (K1622, Reanta Co Ltd). A fluorescent quantitation PCR instrument (ViiA 7, DAAN Gene Co Ltd. of Sun Yat‐sen University) was applied for gene detection. Relative transcription level was calculated using relative quantitative method (2^−∆∆CT^) with 2 μg total cDNA as the template and U6 and β‐actin as the internal references.^19^

**Table 2:** Primer sequences for RT‐qPCR

Gene	Sequence (5'‐3')
miR‐27a	F: TTCACAGTGGCTAAG
	R: GTGCAGGGTCCGAGGT
BTG2	F: GCGCGGGCTCTTCCTCTTTG
	R: AAGGAAGGCTGGAAGAGTGC
β‐actin	F: TGGAGGGGCCGGACTCGTCA
	R: CTTCCTTCCTGGGCATGGAG
U6	F: GCTTCGGCAGCACATATACTAAAAT
	R: CGCTTCACGAATTTGCGTGTCAT

Abbreviations: BTG2, B‐cell translocation gene 2; F, forward; miR‐27a, microRNA‐27a; R, reverse; RT‐qPCR, reverse transcription‐quantitative polymerase chain reaction.

### 2.14 Western blot analysis

Total protein was extracted from both the tissues and cells in strict accordance with the instructions of the efficient radio‐immunoprecipitation assay (RIPA) lysis buffer (R0010, Beijing Solarbio Life Sciences Co Ltd). Next, protein concentration was determined in each sample using a bicinchoninic acid (BCA) kit (20201ES76, Yeasen Biotechnology Co Ltd). Protein transfer onto a polyvinylidene fluoride (PVDF) membrane was performed following isolation by polyacrylamide gel electrophoresis (PAGE) and subsequently sealed with 5% BSA at room temperature for 1 hours. Incubation was subsequently conducted on the membrane with diluted primary rabbit anti‐human antibodies (Abcam Inc) to BTG2 (ab85051, 1:800), vascular endothelial growth factor (VEGF, ab39256, 1:1000), VEGF receptor (VEGFR, ab36844, 1:1000), matrix metalloproteinase‐2 (MMP‐2, ab37150, 1:1000) and MMP‐9 (ab73734, 1:1000) overnight at 4℃. The membrane was then further incubated with HRP‐labelled goat anti‐rabbit IgG diluent (ab205718, 1:20 000, Abcam Inc) at room temperature for 1 hours. ImageJ 1.48 u software (National Institutes of Health) was applied for protein quantitative analysis.

### 2.15 Enzyme‐linked immunosorbent assay (ELISA)

An ELISA kit was employed to determine the VEGF level according to the instructions provided by the kit (Imunbio). The known antigen was diluted by carbonate‐coated buffer (pH = 9.6) into 1‐10 μg/mL and added into a 96‐well plate. Next, 0.1 mL diluent was added to each well and allowed to stand at 4℃ overnight. The reaction wells were incubated with 0.1 mL supernatant of sample in preparation for detection at 37℃ for 1 hours. Blank, negative and positive wells were set as controls. Each well was incubated with 0.1 mL of newly diluted enzyme‐labelled antibody (Abcam Inc) at 37℃ for 35‐40 minutes and developed with 0.1 mL freshly prepared tetramethylbenzidine (TMB) substrate solution (EL0001, InnoReagents Biotechnology Co Ltd) at 37℃ for 10‐30 minutes, followed by the addition of 0.05 mL of 2 mol/L sulphuric acid. A microplate reader (BS‐1101, Detie Experimental Equipment Co Ltd) was then used to determine the optical density (OD) value of each well at an excitation wavelength of 450 nm after the blank control well had been zeroed.

### 2.16 Tumour xenograft in nude mice

A total of 48 female mice with immune deficiency (aged 4‐6 weeks, weighing 18‐21 g, purchased from Shanghai Experimental Animal Center of Chinese Academy of Sciences) were placed on a controlled diet in a specific pathogen‐free (SPF) grade clean shelf with a barrier system. The room was irradiated by ultraviolet light regularly at a room temperature of 24‐26℃ as well as a relative humidity of 40%‐60%. All the cages, padding, drinking water and fodder were all sterilized under high pressure. PANC‐1 cells were transfected with lentivirus with miR‐27a mimic‐NC (lv‐up NC), miR‐27a mimic (lv‐miR‐27a‐up), miR‐27a inhibitor‐NC (lv‐down NC) and miR‐27a inhibitor (lv‐miR‐27a‐down) through the use of a lentivirus transfection reagent, after which the stably transfected cells lines were selected for subsequent experimentation. All the lentivirus transfection reagents were purchased from Shanghai GenePharma Co Ltd.

After 1 week, the pancreas of the female mice was inoculated with stably expressed PANC‐1 cells at the logarithmic growth phase by intraperitoneal injection with 200 μL cell suspension (15 mice per group). After consecutive injections for 3 weeks, the mice were killed, with the tumours collected. A ruler was used to record the minor axis (a) and the major axis (b) of tumours, and the tumour volume was calculated according to the formula: π(a^2^b)/6. The tumour weight was measured. The measurements were repeated 3 times in each group. Finally, peripheral blood was collected to extract serum, followed by the extraction of exosomes from serum in accordance with the above procedure.

### 2.17 Immunohistochemistry for detecting microvessel density (MVD)

Microvessel density was determined in the tumour tissues of the nude mice based on the methods used during immunohistochemistry. The primary rabbit antimouse antibody (sc‐376975) against MVD was purchased from Santa Cruz Biotechnology. The MVD results were evaluated using the Weidner microvessel count method.^20^ Low‐power lens (4 × 10) was first used to observe and find the area with the most microvessels, and medium‐power lens (20 × 10) was utilized to determine number of vessels stained brown. Five power fields were randomly selected from each pathological section to calculate the mean value as MVD. In the event the mean value ≥MVD threshold, vessels were regarded as MVD positive; however, if the result was opposite to the threshold, the vessels were considered to be MVD negative.

### 2.18 Statistical analysis

All experimental statistical analyses were conducted using SPSS 21.0 statistical software (IBM Corp). Measurement data were expressed as mean ± standard. Normal distribution and homogeneity of variance were examined. In the event the data conformed to normal distribution with homogeneity of variance, two groups were compared using unpaired *t* test, while comparisons among multiple groups were analysed by means of one‐way analysis of variance (ANOVA) or repeated measurement ANOVA, and pairwise comparisons within groups were assessed using a post hoc test. Otherwise, rank‐sum test was used. A value of *P* < .05 demonstrated statistical significance.

## 3 RESULTS

### 3.1 miR‐27a might regulate PC by targeting BTG2

**Figure 1:**
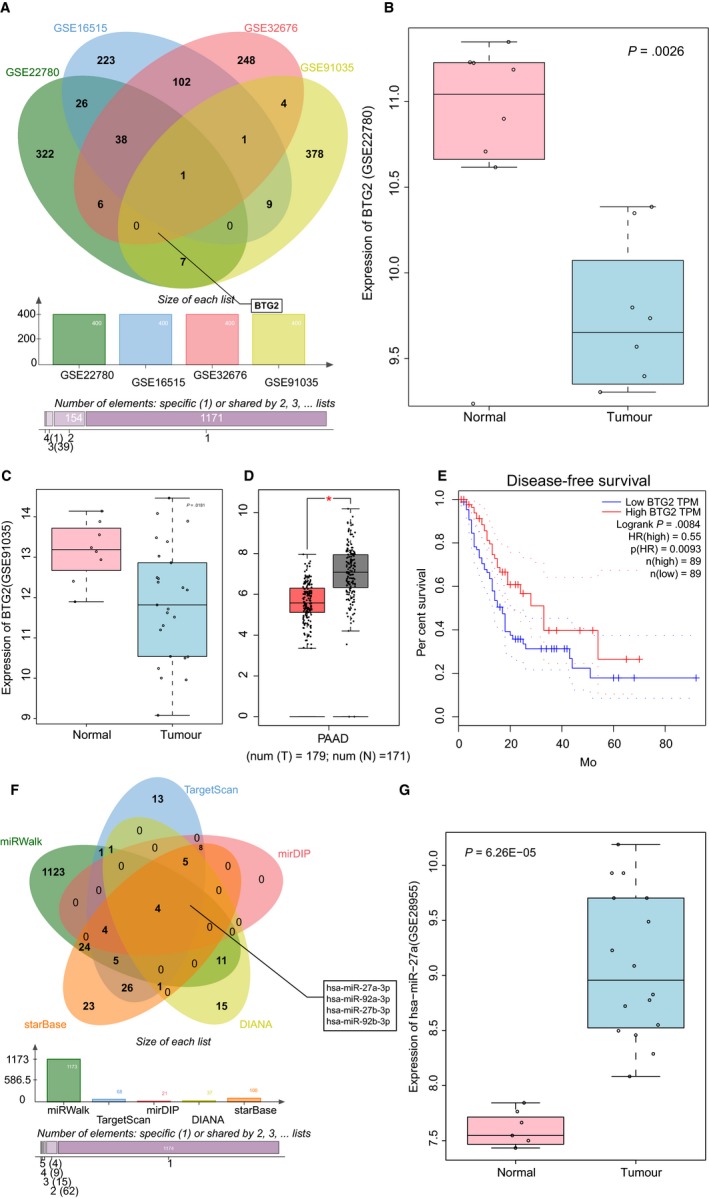
miR‐27a plays a regulatory role in PC via BTG2. A, Comparison results of the top 400 DEGs from http://www.ncbi.nlm.nih.gov/geo/query/acc.cgi?acc=GSE22780, http://www.ncbi.nlm.nih.gov/geo/query/acc.cgi?acc=GSE16515, http://www.ncbi.nlm.nih.gov/geo/query/acc.cgi?acc=GSE32676 and http://www.ncbi.nlm.nih.gov/geo/query/acc.cgi?acc=GSE91035. B and C, BTG2 expression in http://www.ncbi.nlm.nih.gov/geo/query/acc.cgi?acc=GSE22780 and http://www.ncbi.nlm.nih.gov/geo/query/acc.cgi?acc=GSE91035. D, BTG2 expression in PC from GEPIA database. E, The correlation between BTG2 expression and survival of PC patients, as analysed in GEPIA database. F, Comparisons of miRs that regulate BTG2 from miRWalk, TargetScan, mirDIP, DIANA and starBase. G, The expression of hsa‐miR‐27a in http://www.ncbi.nlm.nih.gov/geo/query/acc.cgi?acc=GSE28955. miR‐27a‐3p, microRNA‐27a‐3p; BTG2, B‐cell translocation gene 2; PC, pancreatic cancer; DEGs, differentially expressed genes

R language was applied for analysis of the PC‐related microarray data (http://www.ncbi.nlm.nih.gov/geo/query/acc.cgi?acc=GSE22780, http://www.ncbi.nlm.nih.gov/geo/query/acc.cgi?acc=GSE16515, http://www.ncbi.nlm.nih.gov/geo/query/acc.cgi?acc=GSE32676 and http://www.ncbi.nlm.nih.gov/geo/query/acc.cgi?acc=GSE91035), and the comparison of the top 400 DEGs from these data sets was conducted based on |log2FoldChange| > 1.0 and *P* value < .05 (Table S2). Afterwards, the Venn map was plotted (Figure 1A). Altered BTG2 expression was identified in these four data sets. The expression heat map of the top 80 DEGs from http://www.ncbi.nlm.nih.gov/geo/query/acc.cgi?acc=GSE32676 (Figure S1) and http://www.ncbi.nlm.nih.gov/geo/query/acc.cgi?acc=GSE16515 (Figure S2) revealed lower levels of BTG2 in the PC tissues in comparison with the normal tissues. Based on the expression profile of http://www.ncbi.nlm.nih.gov/geo/query/acc.cgi?acc=GSE22780 and http://www.ncbi.nlm.nih.gov/geo/query/acc.cgi?acc=GSE91035, poor expression of BTG2 was witnessed in the PC tissues (Figure 1B&C). After analysis of GEPIA, low BTG2 levels were identified in the PC tissues (Figure 1D). The survival analysis found that PC patients with lower levels of BTG2 presented poor disease‐free survival (Figure 1E). miRWalk, TargetScan, mirDIP, DIANA and starBase were applied to predict regulatory miRs of BTG2. There were 1173 miRs from miRWalk based on energy < −20, 68 miRs from TargetScan, 21 miRs from miRDIP with the screening threshold set as integrated score > 0.8 and 37 miRs from DIANA according to miTG score > 0.9 (Table S3). Based on the prediction results of the Venn map, there were 4 intersection miRs (hsa‐miR‐27a‐3p, hsa‐miR‐92a‐3p, hsa‐miR‐27b‐3p and hsa‐miR‐92b‐3p), which were regarded as the target sources of BTG2 (Figure 1F). Accordingly, 62 and 172 differential miRs were selected from PC‐related miR microarray data (http://www.ncbi.nlm.nih.gov/geo/query/acc.cgi?acc=GSE41369 and http://www.ncbi.nlm.nih.gov/geo/query/acc.cgi?acc=GSE2895) (Table S4). The differential expression analysis on microarray data revealed that only hsa‐miR‐27a‐3p was highly expressed in PC among 4 regulatory miRs of BTG2. The expression heat map of http://www.ncbi.nlm.nih.gov/geo/query/acc.cgi?acc=GSE41369 is shown in Figure S3, and the expression of hsa‐miR‐27a in http://www.ncbi.nlm.nih.gov/geo/query/acc.cgi?acc=GSE28955 is illustrated in Figure 1G. Based on the aforementioned results, we speculated that miR‐27a could regulate BTG2 in PC.

### 3.2 miR‐27a negatively regulates BTG2 in PC cells

**Figure 2:**
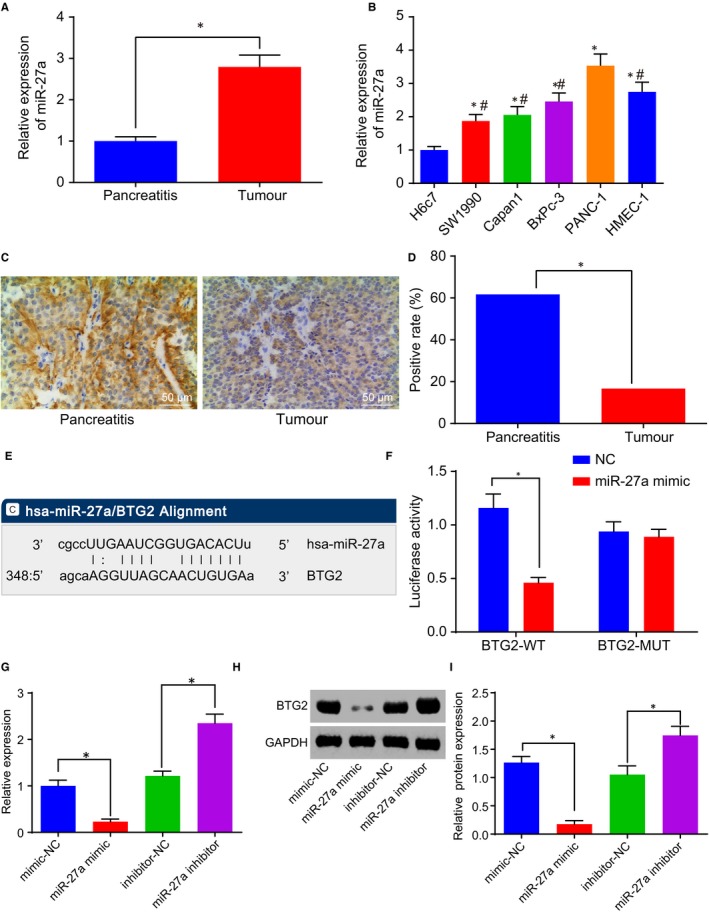
BTG2 is a target gene of miR‐27a. A, miR‐27a expression in PC tissues. B, miR‐27a expression in PC cell lines. C and D, Positive level of BTG2 in PC tissues (200×). E, The binding sites of miR‐27a and BTG2. F, The luciferase activity determination in PANC‐1 cells treated with the reporter plasmids and either miR‐27a or mimic‐NC. G, mRNA level of BTG2 in the presence of miR‐27a mimic or inhibitor. H and I, Protein level of BTG2 in the presence of miR‐27a mimic or inhibitor. *P < .05 vs PANC‐1 cells treated with mimic‐NC or inhibitor‐NC. The above data are measurement data and described as mean ± standard deviation. Panel A is analysed using a paired *t* test, panels B‐I are examined by one‐way analysis of variance. B is enumeration data and checked by *chi*‐square test. The experiment was repeated 3 times independently. miR‐27a, microRNA‐27a; BTG2, B‐cell translocation gene 2; PC, pancreatic cancer; NC, negative control

RT‐qPCR was performed to detect the expression of miR‐27a in PC. The results revealed an overexpression of miR‐27a expression in PC tissues compared with pancreatic tissues obtained from patients with pancreatitis (*P* < .05; Figure 2A). Next, RT‐qPCR was conducted again to determine the expression of miR‐27a in PC cell lines. Overexpression of miR‐27a was detected in the PC cell lines SW1990, Capan‐1, BxPc‐3 and PANC‐1 when compared to the human pancreatic ductal cell line H6c7, with the highest expression identified in the PANC‐1 cells (*P* < .05; Figure 2B). The aforementioned results revealed high expression of miR‐27a in both the PC tissues and cells.

Immunohistochemistry was performed to evaluate the positive level of BTG2 in PC. The results indicated that the positive level of BTG2 was predominately located in the cytoplasm and was brownish in appearance in PC (Figure 2C). BTG2‐positive level was lower in PC tissues than in pancreatic tissues obtained from patients with pancreatitis (*P* < .05; Figure 2D). Online analysis software found a specific binding region between BTG2 and miR‐27a sequences (Figure 2E). To confirm this prediction, dual‐luciferase reporter gene assay, RT‐qPCR and Western blot analysis were performed. The dual‐luciferase reporter gene assay results revealed a reduction in luciferase activity in the cells co‐treated with miR‐27a mimic and PGLO‐BTG2 WT, while the luciferase activity did not differ significantly in the cells treated with miR‐27a mimic and PGLO‐BTG2 MUT, suggesting that miR‐27a could specifically bind to BTG2 (Figure 2F). The RT‐qPCR and Western blot analysis (Figure 2G‐I) results illustrated that elevated miR‐27a led to a decrease in the level of BTG2, and depleted miR‐27a led to increased levels of BTG2. The relationship between the expression of miR‐27a and BTG2 and the clinical manifestations of PC patients was subsequently investigated (Table S1). There was no correlation detected in relation to the expression of miR‐27a and BTG2 with age and gender of PC patients (*P* > .05). The expression of miR‐27a and BTG2 was found to be correlated with differentiation, tumour node metastasis (TNM) stage and lymph node metastasis in PC patients (*P* < .05). Taken together, the results further demonstrated that BTG2 was negatively regulated by miR‐27a.

### 3.3 miR‐27a depletion inhibits proliferation and invasion but promotes apoptosis of PC cells

**Figure 3:**
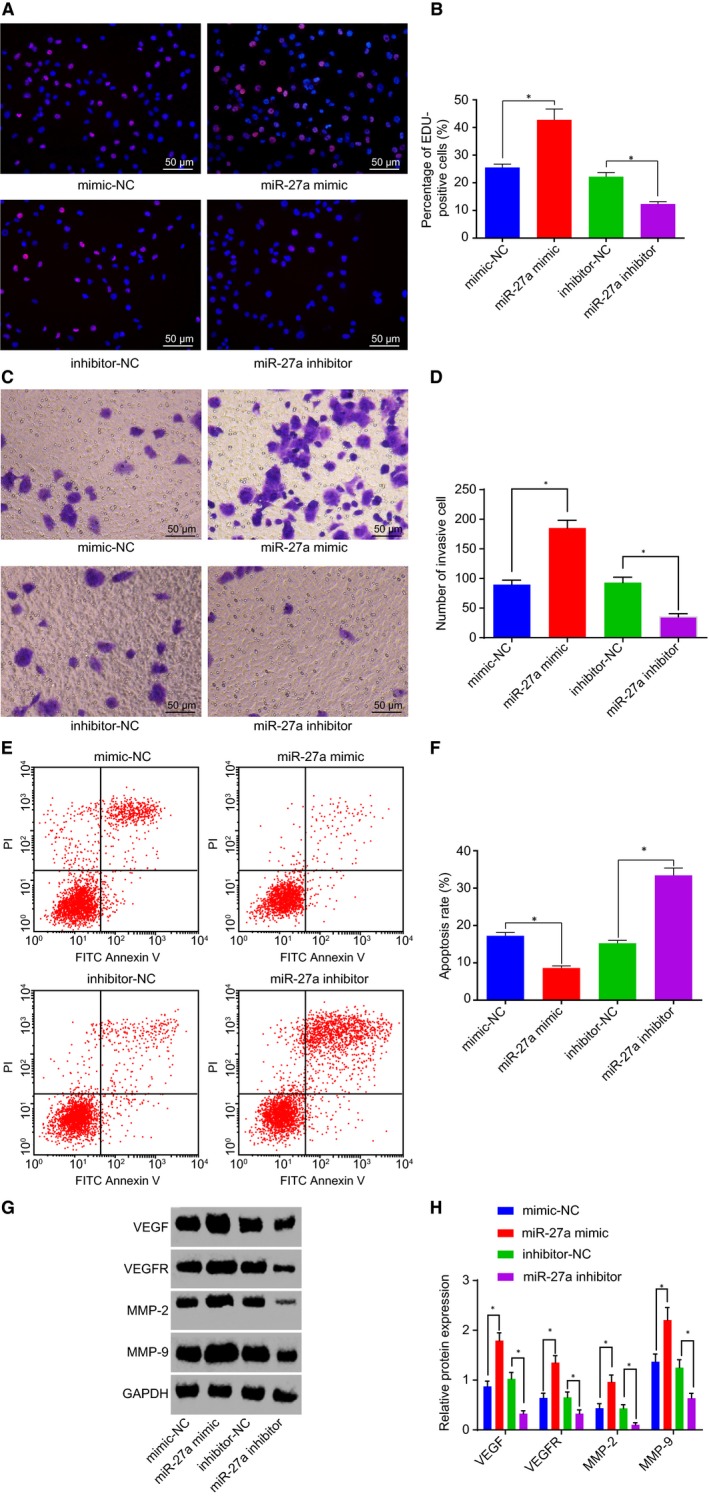
miR‐27a inhibition restrains PC cell proliferation and invasion but accelerates apoptosis. A and B, Positive cell proliferation in PC in response to the treatment of miR‐27a mimic or inhibitor, as detected by EdU (200×). C and D, Cell invasion in PC in response to the treatment of miR‐27a mimic or inhibitor (200×). E and F, Cell apoptosis in PC in response to the treatment of miR‐27a mimic or inhibitor. G and H, Protein levels of VEGF, VEGFR, MMP‐2 and MMP‐9 in response to the treatment of miR‐27a mimic or inhibitor. *P < .05 vs PANC‐1 cells treated with mimic‐NC or inhibitor‐NC. The above data are measurement data and described as mean ± standard deviation. Comparisons among multiple groups are analysed by one‐way analysis of variance. The experiment is repeated three times independently. miR‐27a, microRNA‐27a; PC, pancreatic cancer; NC, negative control; EdU, 5‐ethynyl‐2’‐deoxyuridine; VEGF, vascular endothelial growth factor; VEGFR, vascular endothelial growth factor receptor; MMP, matrix metallopeptidase

The PANC‐1 cells were treated with miR‐27a mimic or inhibitor in order to alter miR‐27a expression. Next, PC cell proliferation, invasion and apoptosis were determined through the application of an EdU assay, Transwell assay and flow cytometry, respectively. The results revealed that inhibitor‐NC and mimic‐NC had no significant effect on proliferation, invasion and apoptosis of PC cells (Figure S4). Also, we observed an increase in red positive cell proliferation and cell invasion but decreased apoptosis in the cells treated with miR‐27a mimic, and a contrasting trend was observed in the cells treated with miR‐27a inhibitor (Figure 3A‐F). Western blot analysis was conducted to detect protein levels of angiogenesis growth factor (VEGF and VEGFR) and migration‐related factors (MMP‐2 and MMP‐9). The results demonstrated an increase in protein levels of VEGF, VEGFR, MMP‐2 and MMP‐9 following the treatment of miR‐27a mimic, which was accompanied by a reduction after treatment with a miR‐27a inhibitor (Figure 3G&H). Thus, miR‐27a down‐regulation was concluded to induce the inhibition of cell proliferation and invasion, and accelerated PC apoptosis.

### 3.4 miR‐27a down‐regulation suppresses cell proliferation and invasion and enhances apoptosis in PC by up‐regulating BTG2

**Figure 4:**
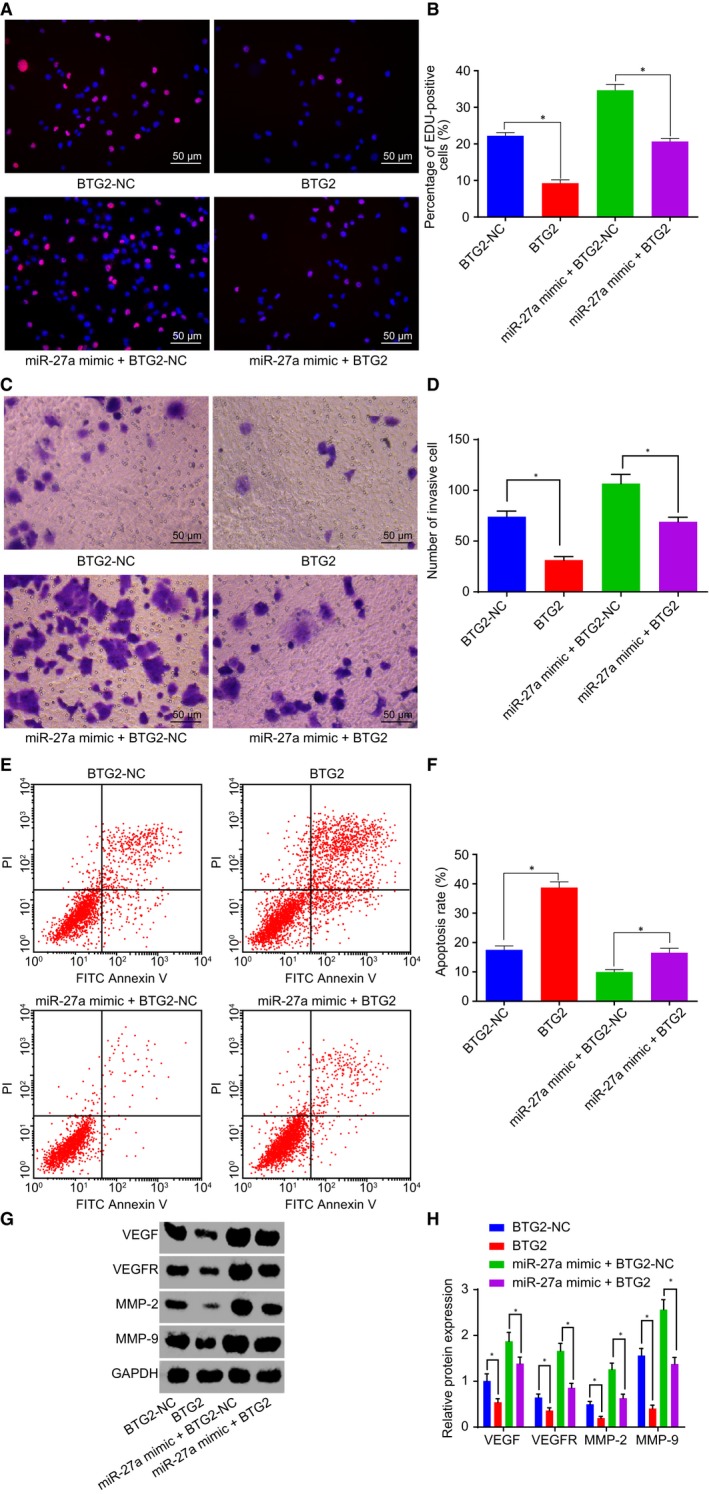
Depletion of miR‐27a up‐regulates BTG2 to restrain cell proliferation and invasion and to promote apoptosis in PC. A and B, Positive cell proliferation in PC after the treatment of BTG2 plasmid and the co‐treatment of miR‐27a mimic and BTG2 plasmid detected by EdU (200×). C and D, Cell invasion in PC after the treatment of BTG2 plasmid and the co‐treatment of miR‐27a mimic and BTG2 plasmid (200×). E and F, Cell apoptosis in PC after the treatment of BTG2 plasmid and the co‐treatment of miR‐27a mimic and BTG2 plasmid. G and H, Protein levels of VEGF, VEGFR, MMP‐2 and MMP‐9 after the treatment of BTG2 plasmid and the co‐treatment of miR‐27a mimic and BTG2 plasmid. *P < .05 vs PANC‐1 cells treated with BTG2‐NC or both miR‐27a mimic and BTG2. The above data are measurement data and described as mean ± standard deviation. Comparisons among multiple groups are analysed by one‐way analysis of variance. The experiment was repeated 3 times independently. miR‐27a, microRNA‐27a; BTG2, B‐cell translocation gene 2; PC, pancreatic cancer; NC, negative control; EdU, 5‐ethynyl‐2’‐deoxyuridine; VEGF, vascular endothelial growth factor; VEGFR, vascular endothelial growth factor receptor; MMP, matrix metallopeptidase

BTG2‐overexpressed plasmids were introduced into the PANC‐1 cells for interference of BTG2 level along with miR‐27a mimic. Next, EdU assay, Transwell assay and flow cytometry were performed respectively to measure PANC‐1 cell proliferation, invasion and apoptosis. The results indicated that the treatment of BTG2 plasmid led to a decrease in red positive cell proliferation and invasion and an enhancement of apoptosis, while these effects were reversed following co‐treatment with miR‐27a mimic and BTG2‐NC (Figure 4A‐F). Western blot analysis was employed to detect the protein levels of VEGF, VEGFR, MMP‐2 and MMP‐9. The results collected indicated that the protein levels of VEGF, VEGFR, MMP‐2 and MMP‐9 were inhibited following the treatment of BTG2 plasmid, and it was promoted by the co‐treatment of miR‐27a mimic and BTG2‐NC (Figure 4G‐H). The aforementioned findings suggested that the down‐regulation of miR‐27a led to an elevation of BTG2, thus inhibiting cell proliferation, invasion and promoting apoptosis in PC.

### 3.5 Depleted miR‐27a inhibits PC occurrence and angiogenesis in vivo

**Figure 5:**
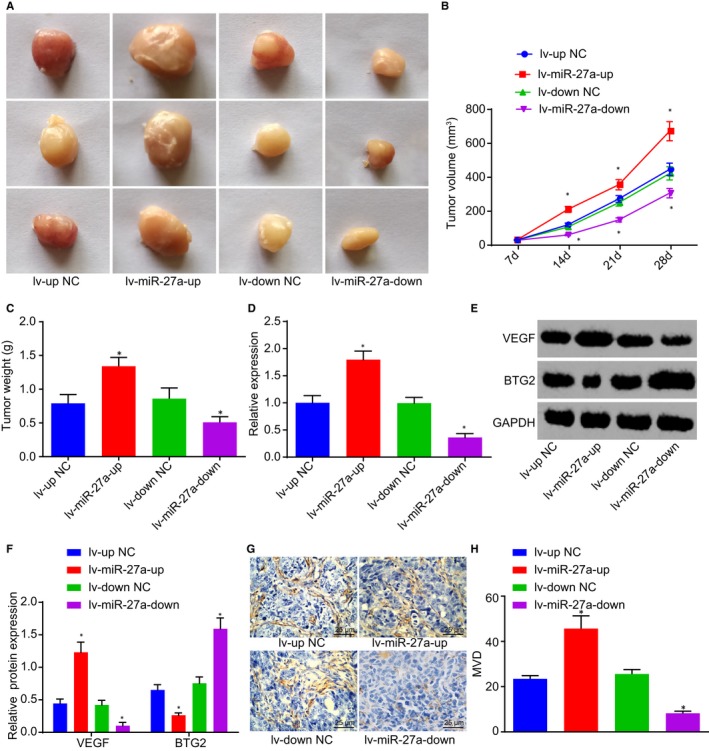
Inhibition of tumour growth and angiogenesis of PC in vivo are induced by miR‐27a silencing. A, Tumorigenesis of PANC‐1 cells following the injection of lentivirus with miR‐27a mimic and miR‐27a inhibitor. B and C, Tumour volume and weight after alteration of miR‐27a. D, The expression of miR‐27a in serum exosomes of nude mice after alteration of miR‐27a. E and F, Protein levels of BTG2 and VEGF in tumour tissues of nude mice after alteration of miR‐27a. G and H, MVD in tumour tissues of nude mice after alteration of miR‐27a (400×). *P < .05 vs nude mice injected with lentivirus with miR‐27a mimic‐NC or miR‐27a inhibitor‐NC. The above data are measurement data and described as mean ± standard deviation. The data of Panel B are analysed by repeated measurement ANOVA; data of other panels are analysed by one‐way analysis of variance. n = 12. The experiment was repeated 3 times independently. miR‐27a, microRNA‐27a; BTG2, B‐cell translocation gene 2; PC, pancreatic cancer; NC, negative control; MVD, microvessel density; VEGF, vascular endothelial growth factor

Following the injection of the PC cell line PANC‐1 into nude mice, tumour formation rate reached 100%. After 2‐4 weeks of model establishment, an evident protrusion was observed in the midsection of the nude mice as well as a hard tumour mass which was detected upon palpation. The nude mice also presented evident emaciation, loss of appetite and listless and stiff limb exercise. The isolated PC xenograft tumour was observed to be oval with an irregular shape, in addition to having an incomplete tumour formation and immersed pancreatic tissues. All tumour tissues were confirmed pathologically. The overexpression of miR‐27a was identified to lead to the enhancement of the tumorigenesis of PANC‐1 cells, in addition to increase tumour volume and weight, and silencing miR‐27a inhibited the tumorigenesis of PANC‐1 cells, accompanied by reduced tumour volume and weight (Figure 5A‐C).

The exosomes were extracted from the serum of nude mice, after which the expression of miR‐27a in the exosomes was detected by RT‐qPCR, the results of which revealed that miR‐27a overexpression led to an up‐regulation of miR‐27a, and miR‐27a depletion was found to down‐regulate miR‐27a (Figure 5D). Western blot analysis was performed in order to determine the protein levels of BTG2 and VEGF in tumour tissues of nude mice, which revealed that enhanced VEGF level and suppressed BTG2 level were induced by miR‐27a elevation, and the opposite trends were caused by miR‐27a silencing (Figure 5E&F). Finally, immunohistochemistry was performed to detect MVD in tumour tissues of nude mice. Brownish cells or cell clusters were observed and regarded as the new vessels. Elevated miR‐27a led to an increase in MVD, and depleted miR‐27a decreased MVD (Figure 5G&H). Thus, the results suggested that silencing of miR‐27a inhibits the tumorigenesis of PANC‐1 cells and reduced MVD in tumour tissues of nude mice.

### 3.6 PC cells deliver miR‐27a into HMVEC via exosomes

**Figure 6:**
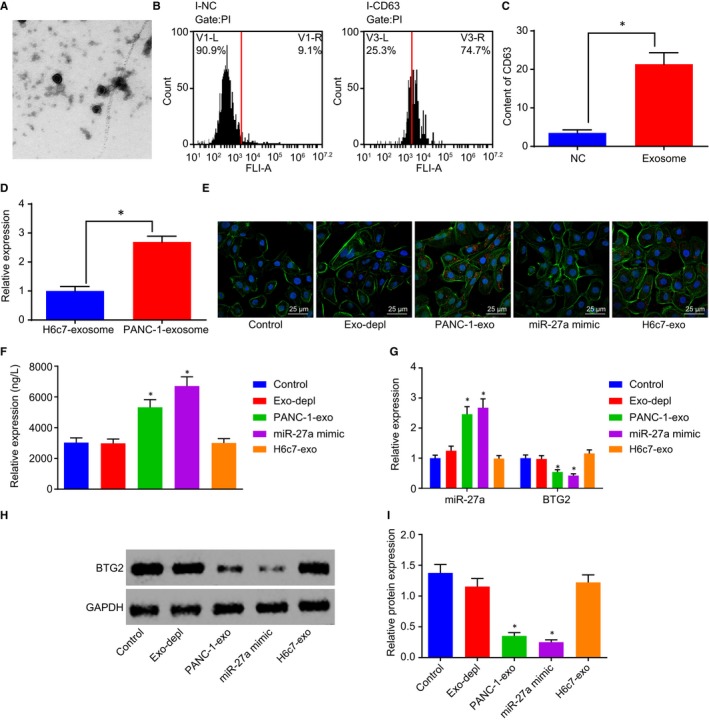
PANC‐1‐exo carries miR‐27a into HMVEC. A, The structure of exosomes identified by TEM (20 000×). B and C, The level of exosome surface marker CD63 in with or without exosomes measured by flow cytometry. D, The expression of miR‐27a in exosomes extracted from H6c7 and PANC‐1 cells. E, The uptake of exosomes by HMEC‐1 cells following the delivery of Exo‐depl, PANC‐1‐exo or miR‐27a mimic (400×). F, VEGF level in the co‐culture medium of PANC‐1‐exo and HMEC‐1 cells following the delivery of Exo‐depl, PANC‐1‐exo or miR‐27a mimic, as measured by ELISA. G, The expression of miR‐27a and BTG2 in HMEC‐1 cells following the delivery of Exo‐depl, PANC‐1‐exo or miR‐27a mimic. H and I, Protein level of BTG2 in HMEC‐1 cells following the delivery of Exo‐depl, PANC‐1‐exo or miR‐27a mimic. *P < .05 vs the treatment without exosomes or with exosomes secreted by H6c7. The above data are measurement data and described as mean ± standard deviation. Comparisons among multiple groups are analysed by one‐way analysis of variance. n = 12. The experiment was repeated 3 times independently. miR‐27a, microRNA‐27a; exo, exosomes; BTG2, B‐cell translocation gene 2; PC, pancreatic cancer; HMVEC, human microvascular endothelial cell; VEGF, vascular endothelial growth factor; TEM, transmission electron microscope; ELISA, enzyme‐linked immunoassay

TEM analysis revealed that the supernatant of PANC‐1 cell culture medium contained exosomes with solid dense bodies, which were generally saucer‐shaped or sphere‐ and vesicle‐shaped with a size of 50‐200 nm (Figure 6A). The flow cytometry was conducted to detect the level of exosome surface marker CD63, the results of which revealed that the exosomes had higher CD63 level, which further indicated that exosomes were successfully extracted (Figure 6B&C). RT‐qPCR was performed in order to detect the expression of miR‐27a in the exosomes extracted from the PANC‐1 cells, which indicated that the expression of miR‐27a was increased in exosomes from PANC‐1 cells in comparison with those from H6c7 cells (*P* < .05; Figure 6D). The uptake of exosomes by the HMEC‐1 cells was analysed under an inverted fluorescence microscope. The results revealed that the delivery of PANC‐1‐exo and H6c7‐exo contained red fluorescence‐labelled exosomes which were not detected following treatment without exosomes or the delivery of Exo‐depl and miR‐27a mimic (Figure 6E). The supernatant of the co‐culture medium and HMEC‐1 cells were separated following a 48‐hours co‐culture of PANC‐1‐exo and HMEC‐1 cells. ELISA was employed to measure VEGF level in co‐culture medium, the results of which revealed there no statistical difference regarding the VEGF level in the treatment without exosomes or the delivery of Exo‐depl (*P* > .05). Compared with the treatment without exosomes, the delivery of PANC‐1‐exo and miR‐27a mimic led to an increased level of VEGF, which was further heightened following the delivery of miR‐27a mimic (*P* < .05; Figure 6F). RT‐qPCR and Western blot analysis were utilized to determine the expression of miR‐27a and BTG2 in HMEC‐1 cells, which exhibited elevated levels of miR‐27a expression along with decreased BTG2 levels following the delivery of PANC‐1‐exo and miR‐27a mimic, whereas the delivery of H6c7‐exo exerted no significant function (Figure 6G‐I). The aforementioned findings suggest that miR‐27a is delivered into HMVEC by PANC‐1‐exo, which further regulates the levels of BTG2 and VEGF.

### 3.7 PANC‐1‐exo carrying miR‐27a promotes HMVEC proliferation, invasion and angiogenesis

**Figure 7:**
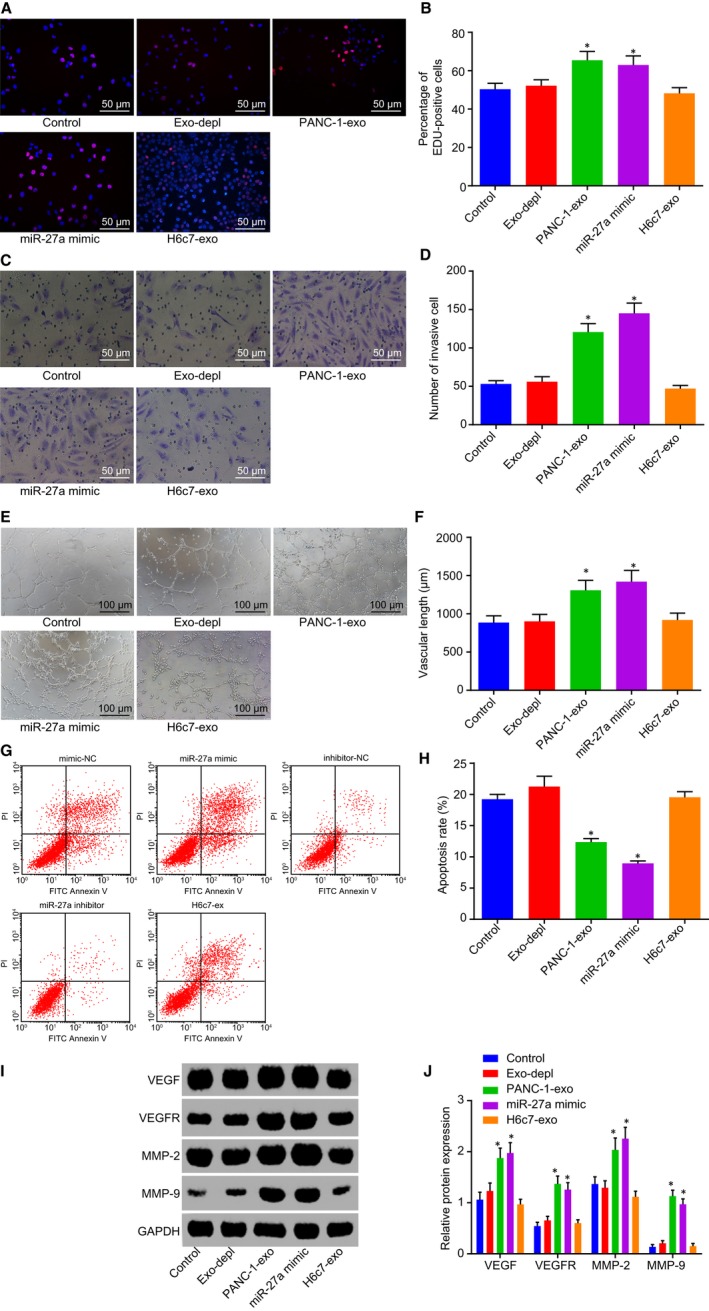
PANC‐1‐exo carrying miR‐27a contributes to acceleration of HMEC‐1 cell proliferation, invasion and angiogenesis but inhibition of apoptosis. A and B, Positive cell proliferation in HMEC‐1 cells in response to the treatment of Exo‐depl, PANC‐1‐exo and miR‐27a mimic detected by EdU (200×). C and D, Cell invasion in HMEC‐1 cells in response to the treatment of Exo‐depl, PANC‐1‐exo and miR‐27a mimic (200×). E and F, Angiogenesis of HMEC‐1 cells in response to the treatment of Exo‐depl, PANC‐1‐exo and miR‐27a mimic (100×). G and H, Cell apoptosis in HMEC‐1 cells in response to the treatment of Exo‐depl, PANC‐1‐exo and miR‐27a mimic. I and J, Protein levels of VEGF, VEGFR, MMP‐2 and MMP‐9 in response to the treatment of Exo‐depl, PANC‐1‐exo and miR‐27a mimic. *P < .05 vs the treatment without exosomes or the delivery of Exo‐depl. The above data are measurement data and described as mean ± standard deviation. Comparisons among multiple groups are analysed by one‐way analysis of variance. The experiment was repeated 3 times independently. miR‐27a, microRNA‐27a; exo, exosome; EdU, 5‐ethynyl‐2’‐deoxyuridine; VEGF, vascular endothelial growth factor; VEGFR, vascular endothelial growth factor receptor; MMP, matrix metallopeptidase

After the co‐culture of PANC‐1‐exo and HMEC‐1 cells, EdU assay, Transwell assay, angiogenesis assay and flow cytometry were performed to detect HMEC‐1 cell proliferation, invasion, angiogenesis and apoptosis. The results revealed there was no statistical difference in cell proliferation, invasion, angiogenesis and apoptosis following the depletion of exosomes in comparison with those without exosome treatment (*P* > .05). The incubation of PANC‐1‐exo and miR‐27a mimic led to an increase in red positive cell proliferation, invasion and angiogenesis and a decrease in apoptosis (Figure 7A‐H). Western blot analysis was performed to determine the protein levels of VEGF, VEGFR, MMP‐2 and MMP‐9, which identified elevated levels of VEGF, VEGFR, MMP‐2 and MMP‐9 following the incubation of PANC‐1‐exo and miR‐27a mimic (Figure 7I‐J). However, no significant changes in red positive cell proliferation, apoptosis, invasion and angiogenesis as well as the protein levels of VEGF, VEGFR, MMP‐2 and MMP‐9 were detected following the co‐culture system of H6c7‐exo and miR‐27a mimic. The aforementioned results suggested that PANC‐1‐exo carrying miR‐27a augments HMEC‐1 cell proliferation, invasion and angiogenesis and reduction in apoptosis.

## 4 DISCUSSION

Despite the progress achieved in the diagnosis and therapy of PC over the past few decades, PC remains one of the most lethal conditions, accompanied by a heavy health burden as well as a high mortality rate.^21^ More recently, cell‐secreted exosomes which are important regulators of cell‐cell communication have been identified as potentially useful tools in gene therapy and drug delivery, and miRs have been reported to be promising therapeutic targets for the treatment of various human diseases.^22^ Hence, the present study investigated the effect of PC cell–derived exosomal miR‐27a on PC through the regulation of BTG2. On the whole, this study suggested that PC cell–derived exosomal miR‐27a knockdown played an inhibitory role in the angiogenesis of HMVEC in PC by up‐regulating BTG2.

One of the important findings from our study revealed that both the PC tissues and cell lines exhibited high expression levels of miR‐27a, accompanied by the poor expression of BTG2. The obtained results suggest that miR‐27a targets and negatively regulated BTG2. Consistent with this finding, Ma Y et al demonstrated that miR‐27a serves as an oncogene in various cancers, and its expression had been found to be up‐regulated in PC.^23^ A recent study concluded that the down‐regulation of miR‐27a results in the inhibition of cell growth and invasion as well as the promotion of apoptosis.^24^ In addition, BTG2 was verified as the target gene of miR‐27a following the detection of luciferase activity and quantification. BTG2 has been reported to be a cancer inhibitor gene and exhibits low levels of expression in tumour tissues, the inhibition of which has been linked with metastasis, invasion and migration of tumour cells.^25^ Meanwhile, a prior study has also provided an insight suggesting that there is a decrease in BTG levels in pancreatic ductal adenocarcinoma, indicating that BTG2 is the direct target of cooperative miR‐2a, miR‐23a and miR‐27a.^14^ All of the aforementioned findings were supporting evidence that miR‐27a negatively regulated BTG2 in PC.

Moreover, our study also revealed that the down‐regulation of miR‐27a and up‐regulation of BTG2 resulted in the inhibition of PC cell proliferation, migration, invasion and angiogenesis and enhancement of cell apoptosis, corresponding to diminished levels of MVD as well as angiogenesis growth factor (VEGF and VEGFR) and invasion‐related factors (MMP‐2 and MMP‐9). VEGF and VEGFR represent important tissue factors for tube formation and the differentiation of angioblast, all of which have been widely reported to play crucial roles in the progression of PC.^26^ VEGF in particular has been shown to be a critical angiogenesis growth factor that facilitates and maintains vascular endothelial cell proliferation and growth.^27^ VEGFs have been shown to have an impact on a large array of cellular processes and initiate the activation of a cascade of downstream signalling pathways, and they do this by interacting with the kinase domain of VEGFRs.^28^ MVD has been correlated with the activity of angiogenesis in tumour formation.^29^ MMPs, which have also been identified as potential cancer biomarkers, have been speculated to facilitate the disintegration of extracellular matrix components with studies implicating their activity in cancer cell metastasis and invasion.^30^ The up‐regulation of both MMP‐2 and MMP‐9, both of which have been shown to play an active role in malignant cell invasion, has been highlighted in PC.^31^, ^32^ Ma Y et al concluded that miR‐27a knockdown contributes to the inhibition of cell growth, migration and colony formation in PC.^23^ In addition, miR‐27a accelerated cancer stem cell differentiation in order to promote angiogenesis in breast cancer.^33^ Elevated levels of BTG2 have been implicated in the inhibition of cell proliferation and invasion, along with the enhancement of apoptosis of MDA‐MB‐231 human triple‐negative breast cancer cells.^34^

The PC cells and HMVEC co‐culture system results revealed that PC cell–derived exosomes delivering miR‐27a act to promote the proliferation, invasion and angiogenesis of HMVEC. There have been multiple cell‐secreted exosomal miRs that have linked to metastasis initiation, resistance to drugs as well as tumour growth.^35^ The co‐culture of exosomes and HMVEC has been found to induce endothelial dysfunction in patients suffering from acute chest syndrome.^36^ Exosomes secreted by cancer cells are widely known with the ability to enter the tumour circulation and microenvironment, highlighting the potential of exosomal miRs as promising biomarkers capable of facilitating the improvement of cancer detection.^37^ The aberrant expression of exosomal miR‐21 has been detected in PC, which may serve as an early diagnostic marker for PC.^38^ Moreover, exosomes containing miR‐27a play a functional role in osteosarcoma with different chemotherapy sensitivity.^39^ The overexpression of exosomal miR‐17‐5p has been linked with metastasis and deterioration of PC into advanced stage.^40^ Exosomes derived from breast cancer MDA‐MB‐231 cells have also been found to promote cell proliferation, invasion and migration in breast cancer.^41^

**Figure 8:**
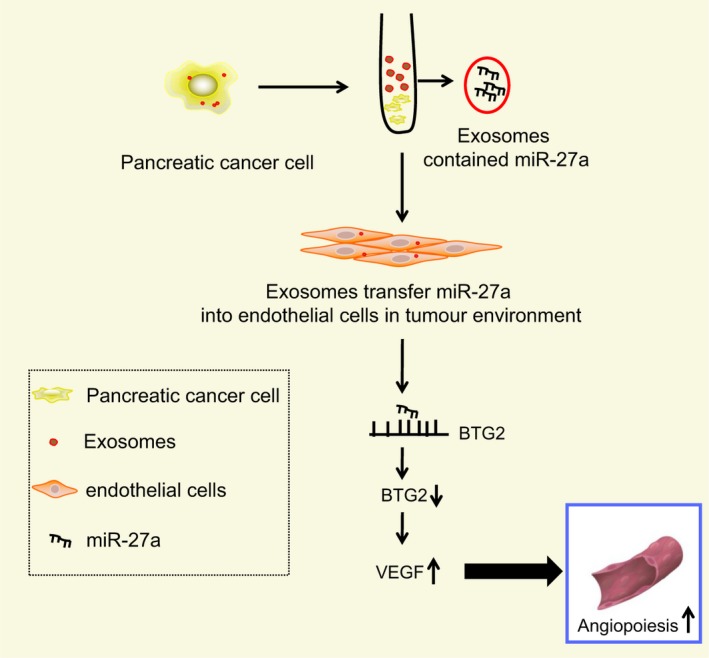
Mechanism of PC cell–derived exosomes containing miR‐27a in angiogenesis of HMVEC in PC. PC cell exosomes transfer miR‐27a into vascular endothelial cells in tumour environment to down‐regulate BTG2 level and up‐regulate levels of VEGF and VEGFR, thus promoting angiogenesis of HMVECs in PC. PC, pancreatic cancer; miR‐27a, microRNA‐27a; HMVEC, human microvascular endothelial cell; BTG2, B‐cell translocation gene 2; VEGF, vascular endothelial growth factor; VEGFR, vascular endothelial growth factor receptor

Taken together, this study indicates that PC cell–derived exosomes carrying miR‐27a have the potential to be a PC therapeutic biomarker due to their promotive effect on the angiogenesis of HMVECs in PC (Figure 8). The aforementioned findings provide evidence that miR‐27a can function as alternative target in therapy of PC. However, owing to the limitation of such objective conditions as time periods and experimental expenditures, future large‐scale studies are required to further elucidate the mechanisms identified in our study, based on which exosomal miRs can be applied to improve treatment outcome of PC.

## CONFLICT OF INTEREST

The authors declare no conflicts of interest.

## AUTHOR CONTRIBUTIONS

DS and CX designed the study. JH collated the data, designed and developed the database. ZSL and ZYY carried out data analyses and produced the initial draft of the manuscript. JRT and YFY contributed to drafting the manuscript. All authors have read and approved the final submitted manuscript.

## Data Availability

The data that support the findings of this study are available from the corresponding author upon reasonable request.

## References

[1] Zhou Y , Shan T , Ding W , et al. Study on mechanism about long noncoding RNA MALAT1 affecting pancreatic cancer by regulating Hippo‐YAP signaling. J Cell Physiol. 2018;233:5805‐5814. 29215734 10.1002/jcp.26357

[2] Shi S , Cao H . Shikonin promotes autophagy in BXPC‐3 human pancreatic cancer cells through the PI3K/Akt signaling pathway. Oncol Lett. 2014;8:1087‐1089. 25120662 10.3892/ol.2014.2293 PMC4114587

[3] Nolan DJ , Ginsberg M , Israely E , et al. Molecular signatures of tissue‐specific microvascular endothelial cell heterogeneity in organ maintenance and regeneration. Dev Cell. 2013;26:204‐219. 23871589 10.1016/j.devcel.2013.06.017 PMC3873200

[4] Cai H , Liu X , Zheng J , et al. Long non‐coding RNA taurine upregulated 1 enhances tumor‐induced angiogenesis through inhibiting microRNA‐299 in human glioblastoma. Oncogene. 2017;36:318‐331. 27345398 10.1038/onc.2016.212

[5] Chiba M , Kubota S , Sato K , et al. Exosomes released from pancreatic cancer cells enhance angiogenic activities via dynamin‐dependent endocytosis in endothelial cells in vitro. Sci Rep. 2018;8:11972. 30097593 10.1038/s41598-018-30446-1 PMC6086824

[6] Kamerkar S , LeBleu VS , Sugimoto H , et al. Exosomes facilitate therapeutic targeting of oncogenic KRAS in pancreatic cancer. Nature. 2017;546:498‐503. 28607485 10.1038/nature22341 PMC5538883

[7] Ding G , Zhou L , Qian Y , et al. Pancreatic cancer‐derived exosomes transfer miRNAs to dendritic cells and inhibit RFXAP expression via miR‐212‐3p. Oncotarget. 2015;6:29877‐29888. 26337469 10.18632/oncotarget.4924 PMC4745769

[8] Zoller M . Pancreatic cancer diagnosis by free and exosomal miRNA. World J Gastrointest Pathophysiol. 2013;4:74‐90. 24340225 10.4291/wjgp.v4.i4.74 PMC3858795

[9] Peng L , Liu Z , Xiao J , et al. MicroRNA‐148a suppresses epithelial‐mesenchymal transition and invasion of pancreatic cancer cells by targeting Wnt10b and inhibiting the Wnt/beta‐catenin signaling pathway. Oncol Rep. 2017;38:301‐308. 28586066 10.3892/or.2017.5705

[10] Li Y , Sarkar FH . microRNA targeted therapeutic approach for pancreatic cancer. Int J Biol Sci. 2016;12:326‐337. 26929739 10.7150/ijbs.15017 PMC4753161

[11] Jutooru I , Chadalapaka G , Abdelrahim M , et al. Methyl 2‐cyano‐3,12‐dioxooleana‐1,9‐dien‐28‐oate decreases specificity protein transcription factors and inhibits pancreatic tumor growth: role of microRNA‐27a. Mol Pharmacol. 2010;78:226‐236. 20488920 10.1124/mol.110.064451 PMC2917860

[12] Zhou L , Liang X , Zhang L , et al. miR‐27a‐3p functions as an oncogene in gastric cancer by targeting BTG2. Oncotarget. 2016;7:51943‐51954. 27409164 10.18632/oncotarget.10460 PMC5239526

[13] Mao B , Xiao H , Zhang Z , et al. microRNA21 regulates the expression of BTG2 in HepG2 liver cancer cells. Mol Med Rep. 2015;12:4917‐4924. 26151427 10.3892/mmr.2015.4051 PMC4581755

[14] Frampton AE , Castellano L , Colombo T , et al. microRNAs cooperatively inhibit a network of tumor suppressor genes to promote pancreatic tumor growth and progression. Gastroenterology. 2014;146(268–277):e218. 10.1053/j.gastro.2013.10.010 24120476

[15] Gautier L , Cope L , Bolstad BM , et al. affy–analysis of Affymetrix GeneChip data at the probe level. Bioinformatics. 2004;20:307‐315. 14960456 10.1093/bioinformatics/btg405

[16] Smyth GK . Linear models and empirical bayes methods for assessing differential expression in microarray experiments. Stat Appl Genet Mol Biol. 2004;3:1‐25. 10.2202/1544-6115.1027 16646809

[17] Atkins D , Reiffen KA , Tegtmeier CL , et al. Immunohistochemical detection of EGFR in paraffin‐embedded tumor tissues: variation in staining intensity due to choice of fixative and storage time of tissue sections. J Histochem Cytochem. 2004;52:893‐901. 15208356 10.1369/jhc.3A6195.2004

[18] Ning H , Albersen M , Lin G , et al. Effects of EdU labeling on mesenchymal stem cells. Cytotherapy. 2013;15:57‐63. 23260086 10.1016/j.jcyt.2012.10.010 PMC3535288

[19] Ayuk SM , Abrahamse H , Houreld NN . The role of photobiomodulation on gene expression of cell adhesion molecules in diabetic wounded fibroblasts in vitro. J Photochem Photobiol B. 2016;161:368‐374. 27295416 10.1016/j.jphotobiol.2016.05.027

[20] Takagi K , Takada T , Amano H , et al. Analysis of microvessels in pancreatic cancer: by light microscopy, confocal laser scan microscopy, and electron microscopy. J Hepatobiliary Pancreat Surg. 2008;15:384‐390. 18670839 10.1007/s00534-007-1241-6

[21] Hamada S , Satoh K , Miura S , et al. miR‐197 induces epithelial‐mesenchymal transition in pancreatic cancer cells by targeting p120 catenin. J Cell Physiol. 2013;228:1255‐1263. 23139153 10.1002/jcp.24280

[22] Zhang D , Lee H , Zhu Z , et al. Enrichment of selective miRNAs in exosomes and delivery of exosomal miRNAs in vitro and in vivo. Am J Physiol Lung Cell Mol Physiol. 2017;312:L110‐L121. 27881406 10.1152/ajplung.00423.2016 PMC5283929

[23] Ma Y , Yu S , Zhao W , et al. miR‐27a regulates the growth, colony formation and migration of pancreatic cancer cells by targeting Sprouty2. Cancer Lett. 2010;298:150‐158. 20638779 10.1016/j.canlet.2010.06.012

[24] Xia J , Cheng L , Mei C , et al. Genistein inhibits cell growth and invasion through regulation of miR‐27a in pancreatic cancer cells. Curr Pharm Des. 2014;20:5348‐5353. 24479798 10.2174/1381612820666140128215756

[25] Lim SK , Choi YW , Lim IK , et al. BTG2 suppresses cancer cell migration through inhibition of Src‐FAK signaling by downregulation of reactive oxygen species generation in mitochondria. Clin Exp Metastasis. 2012;29:901‐913. 22562501 10.1007/s10585-012-9479-z

[26] Costache MI , Ioana M , Iordache S , et al. VEGF expression in Pancreatic cancer and other malignancies: a review of the literature. Rom J Intern Med. 2015;53:199‐208. 26710495 10.1515/rjim-2015-0027

[27] Sun Y , Wu C , Ma J , et al. Toll‐like receptor 4 promotes angiogenesis in pancreatic cancer via PI3K/AKT signaling. Exp Cell Res. 2016;347:274‐282. 27426724 10.1016/j.yexcr.2016.07.009

[28] Aziz MA , Serya RA , Lasheen DS , et al. Discovery of potent VEGFR‐2 inhibitors based on furopyrimidine and thienopyrimidne scaffolds as cancer targeting agents. Sci Rep. 2016;6:24460. 27080011 10.1038/srep24460 PMC4832243

[29] Hansen TF , Sorensen FB , Spindler KL , et al. Microvessel density and the association with single nucleotide polymorphisms of the vascular endothelial growth factor receptor 2 in patients with colorectal cancer. Virchows Arch. 2010;456:251‐260. 20143086 10.1007/s00428-009-0878-8

[30] Lu L , Xue X , Lan J , et al. microRNA‐29a upregulates MMP2 in oral squamous cell carcinoma to promote cancer invasion and anti‐apoptosis. Biomed Pharmacother. 2014;68:13‐19. 24210072 10.1016/j.biopha.2013.10.005

[31] Bu X , Zhao C , Dai X . Involvement of COX‐2/PGE(2) pathway in the upregulation of MMP‐9 expression in pancreatic cancer. Gastroenterol Res Pract. 2011;2011:214269. 21760774 10.1155/2011/214269 PMC3132487

[32] Pan F , Ma S , Cao W , et al. SDF‐1alpha upregulation of MMP‐2 is mediated by p38 MAPK signaling in pancreatic cancer cell lines. Mol Biol Rep. 2013;40:4139‐4146. 23712777 10.1007/s11033-012-2225-4

[33] Tang W , Yu F , Yao H , et al. miR‐27a regulates endothelial differentiation of breast cancer stem like cells. Oncogene. 2014;33:2629‐2638. 23752185 10.1038/onc.2013.214

[34] Zhang YJ , Wei L , Liu M , et al. BTG2 inhibits the proliferation, invasion, and apoptosis of MDA‐MB‐231 triple‐negative breast cancer cells. Tumour Biol. 2013;34:1605‐1613. 23420441 10.1007/s13277-013-0691-5

[35] Sempere LF , Keto J , Fabbri M . Exosomal microRNAs in breast cancer towards diagnostic and therapeutic applications. Cancers (Basel). 2017;9(12):71. 10.3390/cancers9070071 PMC5532607 28672799

[36] Lapping‐Carr G , Khalyfa A , Rangel S , et al. Exosomes contribute to endothelial integrity and acute chest syndrome risk: preliminary findings. Pediatr Pulmonol. 2017;52:1478‐1485. 28486752 10.1002/ppul.23698 PMC5653417

[37] Xu YF , Hannafon BN , Zhao YD , et al. Plasma exosome miR‐196a and miR‐1246 are potential indicators of localized pancreatic cancer. Oncotarget. 2017;8:77028‐77040. 29100367 10.18632/oncotarget.20332 PMC5652761

[38] Goto T , Fujiya M , Konishi H , et al. An elevated expression of serum exosomal microRNA‐191, ‐ 21, ‐451a of pancreatic neoplasm is considered to be efficient diagnostic marker. BMC Cancer. 2018;18:116. 29385987 10.1186/s12885-018-4006-5 PMC5793347

[39] Xu JF , Wang YP , Zhang SJ , et al. Exosomes containing differential expression of microRNA and mRNA in osteosarcoma that can predict response to chemotherapy. Oncotarget. 2017;8:75968‐75978. 29100284 10.18632/oncotarget.18373 PMC5652678

[40] Que R , Ding G , Chen J , et al. Analysis of serum exosomal microRNAs and clinicopathologic features of patients with pancreatic adenocarcinoma. World J Surg Oncol. 2013;11:219. 24007214 10.1186/1477-7819-11-219 PMC3766671

[41] Li XJ , Ren ZJ , Tang JH , et al. Exosomal microRNA miR‐1246 promotes cell proliferation, invasion and drug resistance by targeting CCNG2 in breast cancer. Cell Physiol Biochem. 2017;44:1741‐1748. 29216623 10.1159/000485780

